# Antibiotic practice and stewardship in the management of neutropenic fever: a survey of US institutions

**DOI:** 10.1017/ice.2024.103

**Published:** 2024-10

**Authors:** Xiao Wang, Swarn V. Arya, Sonal Patel, Stephen Saw, Mary A. Decena, Rebecca Hirsh, David A. Pegues, Matthew J. Ziegler

**Affiliations:** 1 Division of Hematology & Oncology, Department of Medicine, University of Pennsylvania, Philadelphia, PA, USA; 2 Division of Hospital Medicine, Department of Medicine, University of Pennsylvania, Philadelphia, PA, USA; 3 Department of Pharmacy, University of Pennsylvania, Philadelphia, PA, USA; 4 Division of Infectious Diseases, Department of Medicine, University of Pennsylvania, Philadelphia, PA, USA

## Abstract

**Objective:**

To describe neutropenic fever management practices among healthcare institutions.

**Design:**

Survey.

**Participants:**

Members of the Society for Healthcare Epidemiology of America Research Network (SRN) representing healthcare institutions within the United States.

**Methods:**

An electronic survey was distributed to SRN representatives, with questions pertaining to demographics, antimicrobial prophylaxis, supportive care, and neutropenic fever management. The survey was distributed from fall 2022 through spring 2023.

**Results:**

40 complete responses were recorded (54.8% response rate), with respondent institutions accounting for approximately 15.7% of 2021 US hematologic malignancy hospitalizations and 14.9% of 2020 US bone marrow transplantations. Most entities have institutional guidelines for neutropenic fever management (35, 87.5%) and prophylaxis (31, 77.5%), and first-line treatment included IV antipseudomonal antibiotics (35, 87.5% cephalosporin; 5, 12.5% penicillin; 0, 0% carbapenem).

We observed significant heterogeneity in treatment course decisions, with roughly half (18, 45.0%) of respondents continuing antibiotics until neutrophil recovery, while the remainder having criteria for de-escalation prior to neutrophil recovery. Respondents were more willing to de-escalate prior to neutrophil recovery in patients with identified clinical (27, 67.5% with pneumonia) or microbiological (30, 75.0% with bacteremia) sources after dedicated treatment courses.

**Conclusions:**

We found substantial variation in the practice of de-escalation of empiric antibiotics relative to neutrophil recovery, highlighting a need for more robust evidence for and adoption of this practice. No respondents use carbapenems as first-line therapy, comparing favorably to prior survey studies conducted in other countries.

## Introduction

Neutropenic fever is a significant source of mortality, morbidity, hospitalizations, and healthcare costs.^1^ Management of neutropenic fever results in prolonged periods of intravenous (IV) broad-spectrum antibiotics, often for the duration of neutropenia. Although multidisciplinary guidelines^2–5^ agree that appropriate prompt treatment of neutropenic fever is critical, the management decisions after initiation of antibiotic therapy are more complex, and guideline recommendations for these decisions have been less clear. This is in the context of increasing recognized harms of unnecessary antimicrobial use, including medication toxicity, healthcare utilization (including cost and length of stay), antimicrobial resistance, and risk of hospital-acquired infections (such as *Clostridioides difficile*).

Specifically, the decision to de-escalate from IV antimicrobial therapy in stable patients with neutropenic fever is of importance. Guidelines generally use an absolute neutrophil count (ANC) goal of 500 cells/uL as a target for stopping IV antibiotics based on a landmark study published in 1979,^6^ though some studies have found no difference in outcomes with lower ANC thresholds for antibiotic cessation.^7^ A growing set of literature has also suggested the safety of de-escalation prior to a dedicated ANC recovery threshold, described in many single- and multi-center retrospective analyses.^8–13^ Indeed, 2023 International Pediatric Fever and Neutropenia Guideline now reflect a recommendation for de-escalation prior to ANC recovery in clinically well and afebrile patients, based a number of randomized controlled trials (RCTs) in this population.^14^

Randomized trials in adults with neutropenic fever are fewer in number, though corroborate the safety of de-escalation prior to ANC recovery. A multicenter RCT from Spain comparing de-escalation after 72 hours of apyrexia versus continuing until ANC recovery, found no difference in adverse events and an expected improvement in antibiotic-free days.^15^ Another recent multicenter RCT in the Netherlands comparing a short (3 days) versus long (9+ days) of carbapenem treatment in neutropenic fever showed no statistical difference in treatment failure, though non-inferiority criterion was not met in the per-protocol treatment comparison.^16^ A third single-center study in cellular therapy patients with neutropenic fever also showed an increase in antibiotic-free neutropenia days with de-escalation after 48 hours of treatment, supported by use of a rapid multiplex polymerase chain reaction assay, with similar rates of breakthrough infections and fevers, 30-day mortality, and cellular therapy-related toxicities.^17^ A recent systematic review of these studies, including both RCT and retrospective findings, showed no statistical difference in mortality, treatment failure (mostly defined by recurrence of fever or clinical infection), or bacteremia comparing short versus long-term duration of antibiotic management in neutropenic fever.^18^

The implementation of strategies to reduce antibiotic exposure in neutropenic fever patients has been studied and described over time via survey studies in various geographical settings (selected surveys displayed in Table 1), primarily conducted in Europe and Asia. Notably, a US-based 2019 survey assessed the presence of institutional guidelines in the management of adult patients with neutropenic fever in US cancer centers.^26^ Here, we report an updated survey of US-based institutions, with an aim to augment and update these data in the setting of increasing evidence supporting antibiotic stewardship prior to neutrophil recovery. Furthermore, we sought to assess and describe real-world management of patients with neutropenic fever rather than institutional guidelines, which may diverge in clinical practice.

**Table 1.** Summary of selected survey studies regarding neutropenic fever management

Study	Location	Survey year(s)	Respondents	NF prophylaxis	NF empiric treatment	Early de-escalation
Yoshida et al. 2004^19^	Japan	2001	125	52% for AML induction	22% cephalosporin alone 13% carbapenem alone 50% beta-lactam + aminoglycoside	Not assessed in survey
Ziglam et al. 2005^20^	UK	2005	167	71% for neutropenia	5% piperacillin-tazobactam alone 72% piperacillin-tazobactam + gentamicin	Not assessed in survey
Choi et al. 2008^21^	Korea	2005–2006	33	42.5% for chemotherapy, 90.9% for SCT	19% cephalosporin alone 50% cephalosporin + aminoglycoside	18.1% de-escalate before NR
Fujita et al. 2009^22^	Japan	2007	134	58% for AML induction	77% cephalosporin alone 31% carbapenem alone 20% cephalosporin + aminoglycoside	Not assessed in survey
Kimura et al. 2017^23^	Japan	2013	141	64% for AML induction	84% cephalosporin alone 16% antipseudomonal penicillin alone 29% carbapenem alone 6% cephalosporin + aminoglycoside	Not assessed in survey
Verlinden et al. 2020^24^	Europe/Asia	2017	194	57.1% (43.7% NW Europe, 71.6% SE Europe, 60.0% Asia)	42% piperacillin-tazobactam alone 20% cephalosporin or carbapenem alone 37% one of above + aminoglycoside	49.5% de-escalate before NR, with unknown source 36.6% (19.9% with complicated case) de-escalate before NR, with positive cultures + susceptibilities 40.9% (21.1% with complicated case) de-escalate before NR, with clinical infection
Kimura et al. 2020^25^	Japan	2019	163	62% for AML induction	79% cephalosporin alone 25% antipseudomonal penicillin alone 19% carbapenem alone 2% cephalosporin + aminoglycoside	27.6% switch to oral antibiotics before NR 19.6% stop antibiotics before NR
Barreto et al. 2022^26^	US	2019	34	87% for high risk patients	90% cefepime alone 66% piperacillin-tazobactam alone 41% meropenem alone	35% with guidelines regarding empiric de-escalation before NR

Note. NF, neutropenic fever; AML, acute myeloid leukemia; SCT, stem cell transplant; NR, neutrophil recovery. Multiple answers allowed for empiric treatment, so percentages may add up to >100%.

## Methods

We developed a survey to assess current clinical practices in neutropenic fever, divided to three sections: demographic information (such as hospital and practice characteristics), antimicrobial prophylaxis and supportive care (including differences based on patient characteristics and screening practices), and neutropenic fever management (including specific antimicrobial choices and clinical scenarios). The survey consisted of 33 questions with some conditional additional questions and took an estimated 10–15 minutes to complete. Of note, the expressed aim of our survey was to assess real-world clinical practice at different institutions, rather than what is recommended by guidelines or by individual respondents. A complete version is included in the Supplementary Materials.

The survey was distributed by e-mail to institutional representatives of US members of the Society for Healthcare Epidemiology of America Research Network (SRN); these representatives include both infectious disease physicians and antimicrobial stewardship pharmacists. Responses were collected in two rounds, from September 2022 to January 2023 (from 45 active original SRN members) and during May 2023 (distributed to 28 new SRN members only). No incentive was provided for respondents. Study data were collected and managed using REDCap electronic data capture tools hosted at the University of Pennsylvania.^27^

## Results

### Respondent characteristics

40 completed total responses were recorded, for a total of 54.8% response rate (24 of 45 in initial round, 16 28 in second round). Characteristics of institutions are reported in Table 2, divided by cohort of respondents (Cohort 1, September 2022 to January 2023; Cohort 2, May 2023). These institutions provide a notable portion of care for cancer patients nationwide, accounting for 16.3% of US hematologic malignancy hospitalizations in 2021 and 15.5% of US bone marrow transplantations in 2020. Most entities reported institutional guidelines for neutropenic fever management (35, 87.5%) and prophylaxis (31, 77.5%). Ten (25.0%) had a dedicated Oncology Infectious Diseases consult service, while another 12 (30.0%) had a consult service that sees all immunosuppressed patients (including oncology, transplant, etc). Most institutions had pharmacists participate in team-based rounds (26, 65.0%). Characteristics of both respondent cohorts were similar, though all pediatric institutions were in the first cohort. Nonetheless, χ^2^ tests of patient population (*P* = .15) and academic setting (*P* = .43) did not show any statistically significant differences.

**Table 2.** Respondent characteristics, overall and by respondent cohort

Characteristic	All respondents	Cohort 1	Cohort 2
**Total respondents**	*N* = 40 (54.8% RR)	*n* = 24 (53.3% RR)	*n* = 16 (57.1% RR)
**Patient population**			
– Adult only	24 (60%)	13 (54.2%)	11 (68.8%)
– Pediatric only	5 (12.5%)	5 (20.8%)	0 (0%)
– Combined	11 (27.5%)	6 (25%)	5 (31.3%)
**Setting**			
– Primary academic	30 (75%)	18 (75%)	12 (75%)
– Academic affiliated	5 (12.5%)	2 (8.3%)	3 (18.8%)
– Non-academic	5 (12.5%)	4 (16.7%)	1 (6.3%)
**Impact of respondents**			
– 2021 US hospitalization volume^a^	16.3%	10.6%	5.7%
– 2020 US BMT volume^b^	15.5%	10.6%	5.0%
– NCI Designated Cancer Center	18 (45%)	10 (41.7%)	8 (50%)
– IDSA Stewardship Center of Excellence	15 (37.5%)	7 (29.2%)	8 (50%)
**Patient volume**			
– Dedicated oncology beds			
– 0 – 29 beds	15 (37.5%)	9 (37.5%)	6 (37.5%)
– 30 – 99 beds	9 (22.5%)	7 (29.2%)	2 (12.5%)
– 100+ beds	7 (17.5%)	4 (16.7%)	3 (18.8%)
– Unknown or not reported	9 (22.5%)	4(16.7%)	5 (31.3%)
– Dedicated malignant hematology beds			
– 0 – 14 beds	7 (17.5%)	2 (8.3%)	5 (31.3%)
– 15 – 49 beds	9 (22.5%)	5 (20.8%)	4 (25%)
– 50+ beds	5 (12.5%)	4 (16.7%)	1 (6.3%)
– Unknown or not reported	19 (47.5%)	13 (54.2%)	6 (37.5%)
**Guidelines & resources**			
– Guideline for oncology prophylaxis present	31 (77.5%)	19 (79.2%)	12 (75%)
– Guideline for neutropenic fever present	35 (87.5%)	20 (83.3%)	15 (93.8%)
– ID routinely consulted for neutropenic fever	9 (22.5%)	6 (25%)	3 (18.8%)
– Oncology ID service present	10 (25%)	5 (20.8%)	5 (31.3%)
– Other immunosuppression ID service present	12 (30%)	7 (29.2%)	5 (31.3%)
– Pharmacists participate in rounds	26 (65%)	16 (66.7%)	10 (62.5)

Note. RR, response rate; BMT, bone marrow transplant; NCI, National Cancer Institute; IDSA, Infectious Diseases Society of America; ID, Infectious Diseases.

a Percentage of 2021 US hospitalizations for hematologic malignancies, based on Centers for Medicare & Medicaid Services records. Diagnosis codes listed in Supplemental Materials.

b Percentage of 2020 US bone marrow transplant cases, based on Health Resources & Services Administration records.

In free text responses regarding general comments about neutropenic fever management, several respondents (n = 7), suggested potential room for improved stewardship in this scenario, such as comments reporting “ID team is much more comfortable de-escalating […] than oncology,” “this is […] very much a negotiation,” “we still have opportunity there to improve de-escalation,” and “[Oncology] team continues to provide [empiric gram negative] coverage despite what the guidelines say.”

### Prophylaxis and screening

Fluoroquinolones were widely used as febrile neutropenia prophylaxis, including 30 (75.0%) for acute myeloid leukemia induction, 26 (65.0%) for acute lymphoblastic leukemia induction, 21 (52.5%) for allogeneic transplant, and 19 (47.5%) for autologous transplant. Trimethoprim-sulfamethoxazole was routinely used in a minority of institutions in various settings (maximum was 7, 17.5% in allogeneic transplant), and no other antibacterial agent was used in more than 3 respondent institutions. Approximately half of institutions (21, 52.5%) routinely screen patients for SARS-CoV-2 prior to chemotherapy. Although there was a numerical difference in this rate between the two survey response sub-cohorts, this did not reach statistical significant (65.2% vs 37.5%, *P* = .12 by χ^2^ test). A small number of institutions screen for vancomycin-resistant enterococci (8, 20.0%), methicillin-resistant *Staphylococcus aureus* (9, 22.5%), and resistant gram-negative organisms (4, 10.0%).

### Initial management of neutropenic fever

ANC cutoff for neutropenia was defined as 500 cells/uL for most respondents (32, 80.0%); six used 1000 cells/uL and two used 200 cells/uL. Cutoff for temperature defining a fever ranged from 37.6°C to 38.5°C (median 38.0°C, mode 38.0°C). Empiric first-line treatment for neutropenic fever is described in Table 3 and primarily included IV antipseudomonal antibiotics (35 cephalosporin, 5 penicillin agent); no respondent administered a carbapenem as first-line treatment of febrile neutropenia. The addition of gram-positive coverage was primarily chosen based on clinical context, and empiric fungal coverage at initiation of neutropenic fever was rare. For patients with prolonged fever (4+ days), there were a variety of options selected for adding or changing antimicrobial agents, including adding vancomycin (15, 37.5%), broadening empiric gram-negative coverage (9, 22.5%), and adding fungal coverage (30, 75.0%). Obtaining cross-sectional imaging, consulting Infectious Diseases, and obtaining daily blood cultures were also selected by a least nine respondents each.

**Table 3.** Management of initial and persistent neutropenic fever

Clinical scenario	Number of institutions
Empiric first-line antibiotic for neutropenic fever	
– Anti-pseudomonal cephalosporin	34 (85.0%)
– Anti-pseudomonal penicillin	5 (12.5%)
Empiric staphylococcal coverage	
– For all cases	3 (7.5%)
– For specific clinical scenarios^a^	
– Skin or line source	25 (62.5%)
– Pulmonary source, without negative MRSA nares	17 (42.5%)
– Central nervous system source	10 (25%)
– Never started empirically	11 (27.5%)
Empiric fungal coverage	4 (10.0%)
Action taken for persistent fever^a,^ ^b^	
– Vancomycin-resistant *Enterococcus* coverage added	3 (7.5%)
– Vancomycin added	15 (37.5%)
– Gram-negative coverage broadened	9 (22.5%)
– Fungal coverage added or broadened	30 (75%)
– Cross-sectional imaging obtained	24 (60%)
– Infectious Diseases consulted	24 (60%)
– Daily blood cultures collected until apyrexia	9 (22.5%)
– No further blood cultures collected	4 (10.0%)

Note. MRSA, methicillin-resistant staphylococcus aureus.

a Multiple selections allowed.

b Scenario described was a stable patient with 4+ days of fever and neutropenia, without culture result or clinical source.

### Ongoing management of neutropenic fever

Six clinical scenarios were presented to survey respondents to assess decisions surrounding de-escalation of antibiotics after initiation (Table 4). For “bland” neutropenic fever (ie, stable patients without a clinical or microbiologic source of infection), roughly half (18, 45.0%) of institutions continue antibiotics until ANC recovery, with the remainder (22, 55.0%) having criteria for de-escalation based on time of apyrexia (16, 40.0%; 5 using 48 hours, 11 using 72 hours) or duration of antibiotics (6, 15.0%). Respondents were more willing to de-escalate prior to ANC recovery in patients with identified clinical (27, 67.5% in pneumonia patient) or microbiological (30, 75.0% in bacteremia patient) sources after dedicated treatment courses.

**Table 4.** De-escalation patient scenarios

Patient scenario	Responses				
Stable patient with **“bland” neutropenic fever** (no clinical source or culture results), clinically improves + defervesces	**Continue** empiric antibiotics until:				
	**ANC recovery**		Set NF duration	Apyrexia 72 hours	Apyrexia 48 hours
	**45%**		15%	28%	12%
Stable patient with **neutropenic fever + pneumonia**, clinically improves + defervesces	**Continue** empiric antibiotics until:				
	ANC recovery		Set NF + pneumonia duration	**Pneumonia duration alone**	
	33%		20%	**47%**	
Stable patient with **neutropenic fever +** ** ***E coli*** bacteremia**, clinically improves + defervesces	**Continue** empiric antibiotics until:				**Narrow** antibiotics until:
	ANC recovery		Set NF + bacteremia duration	Bacteremia duration alone	**Bacteremia duration alone**
	25%		3%	22%	**50%**
Stable patient with **“bland” neutropenic fever** (no clinical source or culture results), **with extended fevers**	**Continue** empiric antibiotics until:				
	**ANC recovery**		Set NF duration + apyrexia	Apyrexia, regardless of duration	Set NF duration, even if still febrile
	**51%**		10%	36%	3%
Patient with neutropenic fever **clinically worsens**, with clinical improvement + apyrexia on **broader antibiotic coverage**	**Continue** empiric broader antibiotics until:			**Switch back** to initial empiric antibiotics until:	
	ANC recovery	**Set NF duration, then prophylaxis**	Set NF duration, then no antibiotics	Set NF duration, then prophylaxis	Set NF duration, then no antibiotics
	18%	**45%**	11%	16%	10%
Stable patient with **recurrent “bland” neutropenic fever** after de-escalation	**N/A**	**Restart** empiric antibiotics until:			**No change:**
	No de-escalation	ANC recovery	Extended NF duration	Set NF duration	**No antibiotics restarted**
	3%	13%	33%	2%	**49%**

Note. NF, neutropenic fever.

“Set NF duration” is in reference to a standard minimum number of days of empiric gram-negative antibiotic treatment for neutropenic fever. Full wording of survey questions and scenarios are provided in Supplemental Materials.

In most cases of de-escalation prior to ANC recovery, prophylactic antibiotics were restarted at that time. In cases where empiric gram-negative coverage was broadened due to clinical instability, this coverage was continued for the duration of therapy. In cases where fever recurred after de-escalation, most respondents were still willing to reattempt de-escalation, though 13 (32.5%) would instead continue treatment until ANC recovery. Only 1 respondent was agreeable with de-escalating if a patient had ongoing fevers. Finally, we asked respondents if a validated biomarker or model for patient outcomes in this population would be helpful to guide de-escalation decisions, with most respondents agreeing to some degree to its benefit (23 agree, 10 neither agree nor disagree, 7 disagree).

## Discussion

Our study is one of only two survey studies of US institutions focused on antibiotic de-decisions during neutropenic fever and the first to focus on real-world management, including practice patterns in specific clinical situations. Our respondents represent a diverse group of institutions while still corresponding to the care of approximately one in six US hematologic malignancy and bone marrow transplant patients, with the infrastructure of SRN resulting in a relatively robust response rate.

Of respondents, most had guidelines for neutropenic fever prophylaxis, with the majority using fluoroquinolones, consistent with clinical guidelines.^4^ Although there are limited randomized data about mortality benefit of antibacterial prophylaxis in neutropenic patients, the clinical benefits in preventing fevers, infections, bacteremia, and hospitalizations are well supported.^28,29^ In addition, a 2005 meta-analysis and 2012 Cochrane review, both done by the same group, show a reduction in all-cause mortality in the use of fluoroquinolones in this setting.^30,31^ However, this benefit is balanced by the potential for resistance; a recent single-center pre/post retrospective study from Italy (in a self-described area of increasing fluoroquinolone resistance) showed a lower rate of fevers but a higher incidence of fluoroquinolone-resistant organisms in leukemia patients receiving prophylaxis, with no difference in blood stream infections or mortality.^32^

No respondents use carbapenems or aminoglycoside combinations as first-line therapy of febrile neutropenia, deviating from what respondents reported in other survey studies in prior years (Table 1). Only four institutions screen for resistant gram-negative bacterial colonization, in contrast to this practice being done more routinely outside of the United States based on increasing proportions of multidrug resistance in the Netherlands,^33,34^ Iran,^35^ Brazil,^36^ Italy,^37^ Spain,^38^ and Japan.^39^ A recent US single-center RCT showed benefit in using ceftolozane-tazobactam over standard-of-care agents for the management of febrile neutropenia.^40^ Despite these reports, a recent analysis of US neutropenic fever cases with bloodstream infections still showed high efficacy of cefepime or piperacillin-tazobactam,^41^ which all of our respondents used as first-line therapy.

We found significant heterogeneity in de-escalation of empiric antibiotics relative to ANC recovery, with varying willingness in doing so in “bland” neutropenic fever, identified clinical infectious source, or microbiologically identified infections. In addition to the previously discussed evidence for de-escalation of empiric antibiotics in this setting,^15,16^ there are some additional limited data about the safety of de-escalation in bloodstream infections, whether to target the culture result^42^ or to treat with a short course of broad antibiotics for low-risk microbes.^43^ This heterogeneity is consistent with the uncertainty in de-escalation reported in the prior US survey study^26^ and highlights a need for more robust evidence for this practice, as well as its education and adoption across US institutions, in the context of local antibiograms and drug resistance.

Of note, even among those institutions with “early” de-escalation protocols, only 1 respondent was comfortable doing since before a period of apyrexia. This practice was consistent with the de-escalation protocol of the How Long trial,^15^ which used a 72-hour period of apyrexia, but was not consistent with the protocol used by de Jonge et al.^16^ The latter randomized one group to de-escalation after 72 hours of carbapenem treatment, regardless of febrile status at the time, compared to a control group of 9+ days of treatment and 5+ days of apyrexia. However, these results did not meet non-inferiority criterion in the per-protocol treatment failure comparison (23% vs 16% in treatment failure, 12% vs 7% in re-admission, and 3% vs 1% in 30-day mortality), with three patients noted to have gram-negative bacillus bacteremia. These findings may explain why our respondent institutions continue to be cautious with de-escalation in patients with continued fevers, despite the trial’s top line findings, as the ongoing fevers likely represent a propensity for treatment failure with recurrent fever, whether from untreated infection or non-infectious fevers from malignant tumor.

Our study provides several notable differences compared to the initial US-based survey reported by Barretto et al.^26^ First, as discussed, we focused on real-world management rather than institutional guidelines, with a focus on specific patient scenarios that capture a diverse number of clinical presentations. Second, although our survey similarly targeted infectious disease physicians and antimicrobial stewardship pharmacists, it was conducted through the SRN, which includes both tertiary referral centers and community-based institutions providing cancer care, rather than focusing solely on cancer centers. Indeed, our response rate compares favorably and captures clinical settings with various levels of academic affiliation and clinical volume. Third, by virtue of its timing, our findings reflect the current state of practice, including any potential impact of the more recent RCT,^16^ as well as the COVID-19 pandemic.

In contrast to these strengths, our study has several limitations as well. Although the use of SRN allowed us to capture a wide variety of healthcare institutions and settings, survey respondents remain limited to SRN members, who have a stated commitment to promote antimicrobial stewardship. This raises the limitation of generalizability to the care of cancer patients in non-SRN institutions, though our response rate was reasonable and compares favorably to the prior US-based study (54.8% vs 30%). Next, the respondents consist of SRN representatives at their institutions, which typically consistent of infectious disease and stewardship providers and pharmacists, rather than hematologists, oncologists, or hospitalists that may be those carrying out direct clinical care for neutropenic fever patients. The decision regarding de-escalation prior to ANC recovery can be complex and nuanced on the “front lines” of direct patient care. Thus, responses about de-escalation may represent a mix of personal clinical judgment, aspirational or tangible institutional guidelines, and a true reflection of clinical decision-making. This is a limitation of most survey studies about stewardship but nonetheless raises challenges with interpretation and comparison between studies. We sought to mitigate these limitations by using concrete clinical scenarios with various situations, which hopefully captures real-world decision-making across a range of potential patient presentations. Furthermore, our findings of the variability of clinical practices, even as reported by stewardship-focused practitioners, reinforces the lack of guideline-directed information to support evidence based uniform practice, rather than simply an implantation issue or differences in cautiousness in clinical practice. Finally, our survey only captured a small number of pediatric healthcare institutions, where guidelines regarding de-escalation are more well established, limiting our ability to draw conclusions about this patient population.

In conclusion, our study provides valuable insight into real-world practice patterns in the management of neutropenic fever at US healthcare institutions, with a robust survey sample accounting for a sizable proportion of oncologic care nationwide. There was relative agreement in the practice of prophylaxis and initial management of neutropenic fever, though with some variation in empiric coverage of non-gram-negative organisms, both upfront and in the case of prolonged fevers. Most notably, we found significant heterogeneity in the de-escalation of empiric gram-negative coverage, representing a potential opportunity for antibiotic stewardship. Though there has been increasing evidence for the safety of this practice, randomized data remain relatively scarce, highlighting the need for more research regarding this question to convince and guide both clinical guidelines and individual practitioners.

## Supporting information

Wang et al. supplementary material Wang et al. supplementary material

## Data Availability

SHEA Research Network provided assistance in data acquisition insofar as survey distribution to member institutions. No other party provided assistance in writing or editing the manuscript.

## Supplementary material

To view supplementary material for this article, please visit https://doi.org/10.1017/ice.2024.103
