# Supplementary material for: Antibiotic practice and stewardship in the management of neutropenic fever: a survey of US institutions
Source: Infect Control Hosp Epidemiol. 2024 Aug 1;45(10):1168–75. doi: 10.1017/ice.2024.103 (PMC11611503; doi:10.1017/ice.2024.103)
Supplement: Wang et al. supplementary material [file S0899823X2400103Xsup001.docx]

Supplemental Table 1. List of Diagnostic Codes for Hospitalization Volume

| Code | Diagnostic Code Description |
| --- | --- |
| 14 | ALLOGENEIC BONE MARROW TRANSPLANT |
| 16 | AUTOLOGOUS BONE MARROW TRANSPLANT W CC/MCC OR T-CELL IMMUNOTHERAPY |
| 17 | AUTOLOGOUS BONE MARROW TRANSPLANT WITHOUT CC/MCC |
| 808 | MAJOR HEMATOLOGICAL AND IMMUNOLOGICAL DIAGNOSES EXCEPT SICKLE CELL C |
| 809 | MAJOR HEMATOL/IMMUN DIAG EXC SICKLE CELL CRISIS & COAGUL W CC |
| 810 | MAJOR HEMATOLOGICAL AND IMMUNOLOGICAL DIAGNOSES EXCEPT SICKLE CELL C |
| 820 | LYMPHOMA AND LEUKEMIA WITH MAJOR O.R. PROCEDURES WITH MCC |
| 821 | LYMPHOMA & LEUKEMIA W MAJOR O.R. PROCEDURE W CC |
| 822 | LYMPHOMA AND LEUKEMIA WITH MAJOR O.R. PROCEDURES WITHOUT CC/MCC |
| 823 | LYMPHOMA & NON-ACUTE LEUKEMIA W OTHER PROC W MCC |
| 824 | LYMPHOMA AND NON-ACUTE LEUKEMIA WITH OTHER PROCEDURES WITH CC |
| 826 | MYELOPROLIFERATIVE DISORDERS OR POORLY DIFFERENTIATED NEOPLASMS WITH |
| 827 | MYELOPROLIF DISORD OR POORLY DIFF NEOPL W MAJ O.R. PROC W CC |
| 828 | MYELOPROLIFERATIVE DISORDERS OR POORLY DIFFERENTIATED NEOPLASMS WITH |
| 829 | MYELOPROLIFERATIVE DISORDERS OR POORLY DIFFERENTIATED NEOPLASMS W OT |
| 834 | ACUTE LEUKEMIA WITHOUT MAJOR O.R. PROCEDURES WITH MCC |
| 835 | ACUTE LEUKEMIA W/O MAJOR O.R. PROCEDURE W CC |
| 837 | CHEMO W ACUTE LEUKEMIA AS SDX OR W HIGH DOSE CHEMO AGENT W MCC |
| 838 | CHEMOTHERAPY WITH ACUTE LEUKEMIA AS SECONDARY DIAGNOSIS WITH CC OR H |
| 839 | CHEMO W ACUTE LEUKEMIA AS SDX W/O CC/MCC |
| 840 | LYMPHOMA AND NON-ACUTE LEUKEMIA WITH MCC |
| 841 | LYMPHOMA & NON-ACUTE LEUKEMIA W CC |
| 843 | OTHER MYELOPROLIF DIS OR POORLY DIFF NEOPL DIAG W MCC |
| 844 | OTHER MYELOPROLIFERATIVE DISORDERS OR POORLY DIFFERENTIATED NEOPLAST |
| 846 | CHEMOTHERAPY WITHOUT ACUTE LEUKEMIA AS SECONDARY DIAGNOSIS WITH MCC |
| 847 | CHEMOTHERAPY W/O ACUTE LEUKEMIA AS SECONDARY DIAGNOSIS W CC |
